# A Web-Based Survey Assessing the Attitudes of Health Care Professionals in Germany Toward the Use of Telemedicine in Pregnancy Monitoring: Cross-Sectional Study

**DOI:** 10.2196/10063

**Published:** 2018-08-08

**Authors:** Niklas Grassl, Juliane Nees, Katharina Schramm, Julia Spratte, Christof Sohn, Timm C Schott, Sarah Schott

**Affiliations:** ^1^ Department of Gynecology and Obstetrics University Women's Clinic Heidelberg Heidelberg Germany; ^2^ Centre of Dentistry, Department of Orthodontics and Orofacial Orthopedics University of Tuebingen Tuebingen Germany

**Keywords:** telemedicine, obstetrics, eHealth, pregnancy monitoring, job satisfaction, sleeping problems, night shift, emergency consultation

## Abstract

**Background:**

The demand for fetal monitoring and constant reassurance is high in pregnant women. Consequently, pregnant women use various health apps and are more likely to visit emergency departments due to subjective but nonurgent complaints. However, electronic health (eHealth) and mobile health (mHealth) solutions are rarely used to prevent nonurgent emergency consultations. To implement modern care solutions, a better understanding of the attitudes, fears, and hopes of health care professionals toward eHealth and mHealth is needed.

**Objective:**

The aim of this study was to investigate the attitudes of health care professionals in obstetrics toward telemedicine.

**Methods:**

A quantitative Web-based survey on health care professionals in obstetrics in Germany was conducted. The participants included nurses, midwives, and physicians of all age groups and job positions working in hospitals that provide various levels of health care. The questionnaire comprised 24 questions about the characteristics of the study population, views about emergency consultations in obstetrics, attitude toward telemedicine, job satisfaction, and sleeping behavior.

**Results:**

In total, 244 health care professionals participated in the Web-based survey. In general, health care professionals were skeptical (170/233, 72.9%) about the use of telemedicine in obstetrics; however, 55.8% (130/233) recognized its potential. Moreover, 72% (62/86) of physicians were optimistic in using apps for pregnancy monitoring, whereas 36.1% (47/130) of nonphysicians (*P*<.001) were not. Significantly, more nonphysicians rejected such developments (75/130, 57.7% rejected) compared with physicians (24/86, 28%; *P*<.001). We also found that obstetricians with more than 10 years of work-experience are more skeptical; however, approximately 49% (18/37) of them believed that telemedicine could reduce nonurgent emergency consultations, whereas 73.2% (106/145) of obstetricians with less than 5 years of experience (*P*=.01) thought otherwise. Our survey revealed a high job satisfaction and a prevalence of regular sleeping problems of 45.9% (91/198) among health care professionals in obstetrics. Surprisingly, both job satisfaction and sleeping problems were independent from the number of night shifts per month (*P*=.77 and *P*=.99, respectively). Yet, 56.6% (112/198) of the survey participants thought they would be happier with their job if they had to work fewer night shifts per month.

**Conclusions:**

Our study reveals an ambivalent attitude toward the use of telemedicine among health care professionals in obstetrics in Germany at the moment. Efforts to promote the use of telemedicine should focus on nurses and midwives because these groups are the most skeptical. By contrast, particularly young physicians recognize the potential of apps in patient care and would like to use such technology in pregnancy monitoring.

## Introduction

### Physician-Patient Interaction in the Digital Age

Information technology is transforming our world and will inevitably change the way physicians monitor their patients’ health. Consequently, the World Health Organization stresses the importance of utilizing information and communication technology opportunities in their global strategy on human resources for health [1]. For telemedicine to reach its full potential, however, it is important to know and understand the attitudes of patients and health care professionals toward such technology.

The general public is open-minded about monitoring their well-being electronically, as illustrated by the fact that 65% of mobile phone users have downloaded at least one health or fitness app and that new devices are being developed to monitor and improve patients´ compliance [2,3]. This does not only apply to a healthy population but also to patients with chronic diseases, for example, kidney transplant recipients [4,5]. However, physicians seem less positive and more reluctant to use electronic health care records and linked Web messaging during patient interaction [6,7]. This partly explains why telemedicine and smart wearable body sensors have not been widely adopted. Instead, their use is limited and oftentimes part of pilot projects [8-11]. At the same time, it also raises the question about the factors that can be used to determine physicians’ attitudes toward telemedicine. Kim et al have found that the intention of physicians to use electronic medical record systems was influenced by performance expectancy and attitude [12]. Therefore, a detailed knowledge about the attitudes and performance expectancies of health care providers toward telemedicine may be essential for a more widespread use of such technologies.

### Telemedicine in Obstetrics

A study on US adults investigating acceptance found that females are more likely to engage in different electronic health (eHealth) behaviors than males [13]. At the same time, younger patient groups tend to be more technophile [14,15], rendering pregnant women extremely fit for the use of telemedicine. Pregnancy requires intense monitoring, especially in the case of high-risk pregnancies. Therefore, the potential of maternal self-monitoring has been recognized for years [16]. Telemonitoring is more cost-effective than traditional patient-physician interaction in high-risk pregnancies [17]. Consequently, a variety of eHealth tools have been developed, ranging from smart wearable sensors for fetal electrocardiography [18,19], health platforms for nutrition and lifestyle during pregnancy [20], to pregnancy-specific mobile phone apps [21]. Pregnant women have a positive attitude toward the use of apps for pregnancy monitoring and improvements in patient empowerment [22].

### Study Aims

These findings raise the question whether the attitude of health care professionals toward the use of such technologies in pregnant women is equally positive. We therefore conducted a Web-based survey among obstetric health care providers investigating their perspective on telemedicine in pregnancy. The questions had a broad scope and covered topics that are related to the introduction of telemedicine, particularly from a health care professional’s perspective, such as perception of emergency consultations, number of night shifts per month, job satisfaction, and sleeping behavior.

### Research Questions

The primary research questions in this survey were as follows:

- What is the attitude of health care professionals toward the surveillance of pregnant women using telemedicine?
- How do physicians and other health care providers differ in terms of attitudes toward telemedicine?
- How is the overall job satisfaction among health care professionals working in labor and delivery?
- How is the sleeping behavior of health care professionals affected by frequent emergency consultations of pregnant women?

## Methods

### Survey Design and Questionnaire

A Web-based open survey addressing health care professionals in obstetrics in Germany investigated their attitudes toward telemedicine. The study population included midwives, nurses, and doctors in training who had completed their specialization in obstetrics and gynecology. We aimed to assess the views of all types of health care professionals in obstetrics and to obtain at least 200 representative responses. The number of responses targeted to achieve sufficient statistical power for subgroup comparison. The study was advertised via email newsletters at the University of Heidelberg, social networks of midwives and doctors in training, and direct distribution of the survey link. Data were collected for 2 months in the spring of 2017 using the survey platform SurveyMonkey. All study participants consented prior to the start of the survey. Furthermore, they agreed that the collected data would be stored for analysis and publication. If no online consent was provided, the potential participants could not access the online questions. The participants did not receive any rewards for participation in this study. One item was displayed per screen, and a completeness check was not implemented. Moreover, the participants could review their answers. The estimated response rate based on the number of health care providers reached by our advertisement was 40%, and the completeness ratio was 80%.

The questionnaire covered four main topics in succession: characteristics of the population, emergency consultations, attitudes toward the use of telemedicine, and sleeping behavior. The questions were made by two experienced obstetricians following literature studies and an interdisciplinary discussion with staff members working in the labor and delivery department of the University of Heidelberg, as well as by a psychologist and a sleep coach. The questions were self-developed and subsequently tested independently by 10 health care professionals from our intended survey population. Their feedback was used to test the questions and prove the reliability of the questionnaire. These test participants were excluded from the final study. This study was approved by the ethics committee of the medical faculty of the University of Heidelberg (S-525/2016).

### Survey Questions

The questionnaire consisted of 22 close-ended questions to appropriately conduct the statistical analysis of the responses. The remaining two questions were open-ended questions, which asked the participants to state their opinion on the use of telemedicine in obstetrics and to provide opinions about the topic in general. The open-ended questions were used as a qualitative account of their attitude toward telemedicine to formulate a hypothesis for the subgroup analyses.

Questions 1-10 consisted of the study population characteristics, such as age, sex, employment, number of years since the completion of training and experience in obstetrics, number of night shifts per month, funding structure of the employing institution, level of care, and number of deliveries per year. Questions 11 and 12 focused on emergency consultations during pregnancy. Questions 13-15 explored the attitudes of health care professionals on the use of telemedicine in general and toward preventing emergency consultations in obstetrics in particular. Questions 14 and 15 referred to a specific scenario: Doctors were asked to imagine that an app that could be used by pregnant women at home to assess for fetal well-being was available. This scenario was developed during an interdisciplinary discussion on health care professionals in the labor and delivery department of the University of Heidelberg to address future care strategies. Questions 15 and 16 examined job satisfaction as well as working conditions of the health care professionals. Finally, questions 18-22 were about sleeping problems as indicators of work-related stress and measures to improve sleeping behavior.

### Statistical Analysis

Statistical analyses were performed using Microsoft Excel version 15.31 and SAS 9.1 Documentation (SAS, Cary, NC). All responses were analyzed even if the 24 questions were not answered completely. The close-ended questions were analyzed by determining the relative frequencies and presented as the percentage of the total number of responses. In some questions, the participants were asked to rank their agreement to a statement on a scale from 1 to 6, in which 1 expressed a strong agreement and 6 a strong disagreement. We analyzed these questions by stating the exact percentages of the responses and by calculating the weighted mean. The hypotheses of the subgroup analyses were formulated based on the responses to the qualitative questions. The medical students were counted as physicians in the comparisons of physicians and nonphysicians. The stated *P* values were determined using chi-square test for categorical data, and the common threshold for a significance level of .05 was used. In cases of questions with rankings from 1 to 6, the weighted means were compared and the *P* values were determined using two-sided *t* tests.

## Results

### Study Population

The characteristics of the study population are presented in Table 1. In brief, 244 health care professionals working in the field of obstetrics in Germany completed the survey. The participants included senior and junior physicians, midwives, nurses, physician assistants, and medical students. Hence, health care professionals from all age groups were represented. Yet, a slight skew toward younger participants was observed, with 60.7% (139/229) of the respondents aged less than 30 years. In accordance with the strong proportion of women among the residents, midwives, and nurses in obstetrics and gynecology, four-fifths of the study population were female (183/229, 79.9%). The majority of physicians were residents (43/78, 55%), which is representative for most hospitals in Germany.

The majority of respondents had several years of experience in obstetrics, of which 28.8% (66/229) had more than 5 years of practice. Furthermore, the median number of births at their workplace was between 2001 and 2500 deliveries/year, and 61.5% (134/218) of the survey participants worked in perinatal centers level I. Perinatal centers of this level offer the most comprehensive treatment, taking care of pregnant women at any gestational age, those with high-risk pregnancy, or those with twin pregnancies [23]. Quite in line with this, slightly more than half of the respondents worked at university hospitals, followed by almost a quarter working in other forms of public hospitals. More than four-fifths (185/228, 81.1%) of the participants worked during night time, mostly between 1 and 4 shifts per month.

### Current Job Situation and Physician Well-Being

This study aimed to explore the working conditions of health care professionals in obstetrics and gynecology and how telemedicine might be able to affect or even improve these working conditions. Table 2 shows the record of obstetricians’ well-being and satisfaction. In summary, the majority of study participants was satisfied with their current working situation and enjoyed working in the obstetrics department. Yet, a minority believed that their work-life balance was good. Surprisingly, however, only one-third of the respondents were in favor of reducing their work load or the number of night shifts. Meanwhile, 56.6% (112/198) of the participants agreed that they would be happier and more satisfied if they had to work fewer night shifts per month.

### Sleeping Behavior

Many health care professionals in obstetrics complained of sleeping problems. Only 16.0% (32/200) of the respondents denied having any sleeping problems. The most common symptoms were tiredness after waking up and tiredness during the day. Approximately 40.1% (77/192) of the respondents experienced these symptoms several times a week. 

**Table 1:** Characteristics of the study population (N=229).

Characteristics		n (%)
**Age (years)**		
	<30	139 (60.7)
	30-35	43 (19.0)
	36-40	19 (8.0)
	41-50	17 (7.0)
	>50	11 (5.0)
**Gender**		
	Female	183 (79.9)
	Male	46 (20.0)
**Job position**		
	Resident in the obstetrics/gynecology department	43 (19.0)
	Consultant	11 (5.0)
	Senior physician	20 (9.0)
	Chief physician	4 (2.0)
	Midwife	36 (16.0)
	Student midwife	66 (29.0)
	Nurse	11 (5.0)
	Student nurse	5 (2.0)
	Physician’s assistant	3 (1.0)
	Medical student	19 (8.0)
**Number of years since the completion of residency**		
	<2	10 (13.0)
	2-5	6 (8.0)
	5-10	6 (8.0)
	>10	9 (12.0)
	Not completed	47 (60.0)
**Years of experience in obstetrics care**		
	<1	58 (25.0)
	1–5	86 (38.0)
	5-10	31 (13.0)
	>10	35 (15.0)
	No experience (students)	19 (8.0)
**Type of employment**		
	Full-time	175 (76.4)
	Part-time	35 (15.0)
	Currently not employed	19 (8.0)
**Night shifts per month**		
	1-4	111 (48.5)
	>5	74 (32.0)
	None	43 (19.0)
**Funding body**		
	University hospital	114 (52.3)
	Public, not a university hospital	51 (23)
	Nonprofit carrier hospital	36 (17.0)
	Private hospital	4 (2.0)
	Others	13 (4.0)
**Care level**		
	Perinatal center level I	134 (61.5)
	Perinatal center level II	20 (9.0)
	Institution that specializes in perinatal care	8 (4.0)
	Basic	23 (11.0)
	Others	33 (15.0)
**Number of births in hospitals per year**		
	500-1000	9 (4.0)
	1001-1500	23 (11.0)
	1501-2000	23 (11.0)
	2001-2500	107 (49.0)
	2501-3000	27 (12.0)
	>3000	10 (5.0)
	Others	19 (9.0)

**Table 2:** Attitude toward their current job situation (N=200).

Statements	Scale^a^, n (%)						Weighted mean (SD)	
	1	2	3	4	5	6		
I am happy with my working situation.	18 (9.0)	75 (37.5)	65 (32.5)	26 (13.0)	10 (5.0)	6 (3.0)		2.8 (1.1)
I would be happier in my job if I had to work fewer night shifts.	50 (25.0)	32 (16.0)	30 (15.0)	13 (6.5)	27 (13.5)	46 (23.0)		3.4 (1.9)
I would like to reduce my average working hours.	36 (18.0)	28 (14.0)	39 (19.5)	39 (19.5)	24 (12.0)	32 (16.0)		3.4 (1.7)
I enjoy working in obstetrics.	101 (50.5)	65 (32.5)	20 (10.0)	8 (4.0)	2 (1.0)	3 (1.5)		1.8 (1.0)
I would like to work fewer night shifts.	40 (20.0)	23 (11.5)	31 (15.5)	48 (24.0)	16 (8.0)	37 (18.5)		3.5 (1.7)
My work life balance is good.	11 (5.5)	30 (15.0)	50 (25.0)	67 (33.5)	24 (12.0)	16 (8.0)		3.6 (1.3)

^a^The participants were asked to indicate their agreement on a scale from 1 to 6, where 1 indicates strong agreement and 6 a strong disagreement.

Only few participants had difficulty falling asleep, which was defined as requiring more than 30 minutes to fall asleep and having trouble staying asleep. By contrast, only few participants regularly presented with muscle cramps or nocturia. The complaints that the study participants experienced as most bothersome were tiredness after waking up (46/200, 23.0%) and extreme tiredness during daytime (40/200, 20.0%), followed by trouble falling asleep (36/200, 18.0%) and staying asleep (30/200, 15.0%).

Consequently, more than half of the study participants (106/196, 54.1%) had already tried to improve their sleeping habits. The most common and most successful attempt to improve sleeping habits was engaging in sports. This improved sleep of two-thirds of those who tried (21/51, 41%). Other, less successful attempts included a change in activities before going to bed (tried by 102/196, 52.0%) and during the day (tried by 93/196, 47.4%), stress and anxiety reduction techniques (tried by 97/196; 49.4%), changes in sleeping time (tired by 99/196, 50.5%), changes in the sleeping environment (tried by 63/196, 32.1%), and the use of medications (tried by 31/196, 15.8%).

### Job Satisfaction in Different Subgroups

We also found that overall job satisfaction of the participants was good and significantly higher in physicians than in nonphysicians (2.9 vs 2.6, *P*=.026). Yet, the physicians were extremely in favor of reducing the number of night shifts and believed that this would increase their job satisfaction. Meanwhile, most nonphysicians did not have the same view (2.4 vs 4.2, *P*<.001). These differences could not be explained by the varying prevalence of sleeping problems between these two groups (51% of nonphysicians vs 54% of physicians, *P*=.77). Our data did not suggest that a reduction of night shifts indeed increased job satisfaction. Health care professionals working 5 or more night shifts per month did not significantly differ in terms of job satisfaction compared with their colleagues who were working at most 4 night shifts per month (2.8 vs 2.7, *P*=.70). Similarly, job satisfaction did not significantly differ between health care professionals working full-time compared with those working part-time (2.9 vs 2.7, *P*=.47). Older study participants (over 40 years) did not have more significant sleeping problems than the younger participants (less than 40 years; 53% vs 46%, *P*=.99). Also, our data did not indicate a correlation between the number of night shifts per month and the prevalence of sleeping problems. Regardless of whether the participants worked night shifts, 46.0% (92/200) regularly experienced sleeping problems (*P*=.99).

### Emergency Consultations and Telemedicine

More than two-thirds of the participants agreed that most emergency consultations were unnecessary. Yet, approximately 72.8% (153/210) still considered such consultations as valid if the patients were worried and insecure about a medically harmless condition. We also asked the study participants to specify the most common reasons for emergency consultations based on their experience (naming multiple reasons was allowed). Besides pain (174/210, 82.8%), bleeding (151/210, 71.9%), labor (153/210, 72.8%), and rupture of membranes (141/210, 67.1%), uncertainty about the well-being of their pregnancy (141/210, 67.1%), negative feelings (84/210, 40.0%), and anxiety or concerns for the baby (78/210, 37.1%) were highly prevalent. By contrast, emergency visits due to reduced fetal movements (80/210, 38%), cervical insufficiency (34/210, 16.2%), acute hypertension (19/210, 9.0%), or trauma (34/210, 16.2%) were less prevalent. In the light of these results, the perspectives of health care professionals on telemedicine may seem particularly relevant because telemedicine promises to prevent some patients from consulting doctors or emergency departments unnecessarily. A majority (143/210, 68.1%) of the study participants agreed that telemedicine would affect unnecessary medical consultations or over consultation.

### Attitudes Toward the Use of Apps in Pregnancy Monitoring

To investigate the attitude of health care professionals toward the use of telemedicine in obstetrics in more detail, we focused on one particular implementation. Namely, we described the scenario in which pregnant women should consult an app when feeling unwell or experiencing unspecific symptoms. This app would then execute a series of measurements and obtain a conclusion from the joint evaluation of the indicated symptoms and measurements. This conclusion could provide either an advice to consult a physician or to give an all-clear without the need to see a doctor.

The attitudes of health care professionals toward this form of telemedicine are listed in Table 3. Surprisingly, 23.9% (56/234) of the respondents reported that they did not understand the concept. Several of them categorically rejected the use of telemedicine in obstetrics. Approximately 72.6% (170/234) had doubts about these new developments, whereas more than half believed in the potential of telemedicine. Few study participants would recommend this form of telemedicine to their patients, and only 37.6% (88/234) were enthusiastic about these developments and were willing to put them into practice as soon as possible.

### More Experienced Participants are Less Confident in Using Telemedicine

In the second step, we analyzed how the attitudes of the health care professionals differed between the subgroups. First, we compared the difference between health care professionals with more than 10 years of experience from those with less than 5 years of experience. The more experienced professionals were significantly less confident in using telemedicine, which could have had an influence on unnecessary night shift consultations, than their less experienced colleagues (49% vs 73.2%, *P*=.01). This difference in terms of attitude might in part be explained by age. We found that only 54% (15/28) of the participants aged greater than 40 years believed that the use of telemedicine results in a change in night shift consultations compared with approximately 76.1% (105/138) of participants aged below 30 years who believed otherwise (*P*=.02). However, the evaluation of the necessity of most night shift consultations was similar across all age groups and level of experience.

**Table 3:** Attitude toward the surveillance of pregnant women using telemedicine (N=234).

Statements	Scale^a^, n (%)						Weighted mean (SD)
	1	2	3	4	5	6	
I do not understand the concept.	28 (12.0)	28 (12.0)	57 (24.3)	40 (17.1)	51 (21.8)	30 (12.8)	3.6 (1.6)
I reject such developments categorically.	26 (11.1)	33 (14.1)	40 (17.1)	47 (20.0)	40 (17.1)	47 (20.0)	3.8 (1.6)
I have my doubts about these developments.	49 (20.9)	53 (22.6)	68 (29.0)	30 (12.8)	16 (6.8)	17 (7.3)	2.8 (1.5)
I see potential in these developments.	26 (11.1)	44 (18.8)	60 (25.6)	60 (25.6)	25 (10.7)	18 (7.7)	3.3 (1.4)
I would recommend such developments to my patients.	18 (7.7)	49 (20.9)	42 (17.9)	56 (23.9)	34 (14.5)	34 (14.5)	3.6 (1.5)
I find such developments excellent and would like to put them into practice as soon as possible	18 (7.7)	26 (11.1)	44 (18.8)	56 (23.9)	33 (14.1)	56 (23.9)	3.5 (1.6)

^a^The participants were asked to indicate their agreement on a scale from 1 to 6, where 1 indicates a strong agreement and 6 a strong disagreement.

**Table 4:** Differences in the attitudes between physicians and nonphysicians (N=218).

Statements		Physicians (n=89)	Nonphysicians (n=129)	Statistics		*P* value
				χ^2^_2_	*t* test^a^	
I consider the use of apps for pregnancy monitoring reasonable, n (%)		62 (69.7)	47 (36.4)	15	—	<.001
I reject such developments categorically, n (%)		24 (27.0)	75 (58.1)	21	—	<.001
How satisfied are you in your job^b^, mean (SD)		2.6 (1.1)	2.9 (1.0)	—	2.2	.03
Would you be happier in your job if you had to work fewer night shifts^b^, mean (SD)		2.4 (1.2)	4.2 (1.2)	—	11	<.001
Do you experience sleeping problems, n (%)		47 (52.8)	66 (51.2)	0.09	—	.77

^a^Two-sided *t* test.

^b^1=very; 6=not at all.

### Physicians are More Open and Optimistic Toward Telemedicine Than Nonphysicians

The attitudes toward the use of apps for pregnancy monitoring diverged substantially between physicians and nonphysicians, such as midwives and nurses (Table 4). Moreover, 72% (64/89) of physicians believed that the development of such apps is reasonable, whereas only 36.1% (47/130) of nonphysicians thought the same way (*P*<.001).

Consequently, 57.7% (75/130) of nonphysician health care professionals categorically rejected such developments compared with just 27% (24/89) of physicians who did not (*P*<.001). The following reasons were frequently associated with rejection: fear that actual emergencies are detected too late, lack of personal care for worried patients, and fear of replacement of human workforce by machines. On the contrary, those in favor of such technologies hoped that this form of telemedicine would reduce unnecessary emergency consultations and allow health care professionals to focus on actual emergencies. No significant differences were observed in the regression analysis of demographic variables, such as sex, job position among physicians, sleeping behavior, and job satisfaction.

## Discussion

### Principal Findings

This is the largest survey on the attitudes of health care professionals in obstetrics in Germany toward telemedicine. Our study showed that the majority of health care professionals in obstetrics perceived most emergency consultations as unnecessary, indicating the need for new strategies to overcome this added workload. The study participants with several years of experience were skeptical about the use of telemedicine to avoid unnecessary consultations, whereas younger, less experienced colleagues were more optimistic in this regard. Interestingly, we found a significant difference in the attitudes toward telemedicine between physicians and nonphysicians. Physicians were more open and optimistic about the potential of telemedicine than other health care providers. Furthermore, despite the limited use of telemedicine and the fact that approximately 40% of the study participants (n=77) presented with sleeping disorders, the overall job satisfaction was high, regardless of age and the number of night shifts per month.

### Comparison With Prior Work

These results are in accordance with those of other previous studies, showing that doctors are satisfied with their profession in general [24]. However, this is usually affected by organizational issues both in Germany [25] and other countries worldwide [26]. Recent studies have indicated that the workload of most doctors in Germany increased within the last couple of years [27] and that most health care professionals are aware of the health risks associated with night shifts, such as sleeping problems and hypertension [28-30], and this result is in accordance with the responses in our survey. A recent study has found that faculty members are dissatisfied with working night shifts compared with residents and emergency medicine nurses [31]. Furthermore, they abstained more often from pharmacological sleeping aids and had a higher rate of accidents while driving home [31].

Although there is no evidence that flexibility in duty hours has an impact on patient outcomes, education quality or resident satisfaction [32], a relevant proportion of physicians in Germany considers that personal consequences are associated with increased workload and the high pace in patient care [33]. These indicators are alarming considering the demographic development and existing shortage of physicians in Germany. Consequently, innovative solutions are crucial to meet these challenges and maintain high-quality health care. Telemedicine promises home-based pre-evaluation of patients and may substantially decrease the workload in a variety of medical specialties. Our study suggests that this potential is increasingly recognized by physicians.

### Outlook Toward Telemedicine in Obstetrics

Emergency departments are increasingly used for nonurgent visits in general and in pregnancy-related concerns in particular, both in the United States and Germany [34,35] (Schramm et al under review). This is unsatisfactory given that addressing nonurgent problems in outpatient settings is more cost-effective and that it leads to a higher quality of life for patients [35]. Our study highlighted that health care professionals in obstetrics in Germany are well aware of this problem. Yet, despite the potential of telemedicine to alleviate this problem, it has not been widely adopted in obstetrics [8,30,32-34]. This is contrasted by a growing demand for Web-based pregnancy apps among patients [22] and a general interest in health care apps [5,15,36]. It is also surprising because other countries and specialties, such as internal medicine or neurology, have already adopted telemedicine in patient care [37-39].

Our survey suggests that especially nonphysicians are still not convinced of the benefits and potential of telemedicine for pregnancy monitoring. The regression analysis of other demographic characteristics did not obtain significant results, probably due to insufficient statistical power in the respective subgroups. Hence, larger scaled studies are needed to evaluate possible differences between these subgroups.

The majority of nurses and midwives seemed to reject telemedicine partly because they were skeptical about the replacement of human labor by machines or intelligent algorithms. Therefore, health care professionals must be reassured that telemedicine is not a threat to their work and that it could help in allowing them to focus on patients who urgently need help. This is of particular importance given the increasing shortage of midwives in obstetrics care [40]. One should also bear in mind that our study included only 33 (16%) midwives. Hence, studies including more professionals are needed to support these preliminary results. However, the number of participants in this study was in concordance with other studies in this field [25,41].

The use of silver bullet in establishing wider acceptance of telemedicine in obstetrics care aimed to develop smart wearable devices that offer practical and reliable monitoring services for pregnant women. These technologies could facilitate the empowerment of mothers to autonomously assess fetal well-being to some degree at home. Ideally, this implementation could help reduce the burden in emergency departments and could improve the standard of care through closer pregnancy observation at the same time. The willingness of expectant mothers to engage in these forms of pregnancy monitoring is well established [22]. At the same time, self-monitoring of blood pressure in individuals with hypertension significantly reduces blood pressure in conjunction with cointerventions [42]. Similarly, self-care in individuals with heart failure via telemonitoring improves their quality of life [43]. The eHealth literacy among women of reproductive age is more favorable than that among other patients who usually use telemedicine [44-47]. Consequently, the development and application of telemonitoring in obstetrics care should be implemented more vigorously and decisively.

### Conclusions

Our study reveals that an ambivalent attitude toward the use of telemedicine in pregnancy monitoring prevails among the health care professionals working in obstetrics in Germany. Frequent health education must be provided to improve skepticism and insecurity among health care professionals, especially among midwives and nurses. Given the enthusiasm we found among young doctors and the openness of patients who are pregnant toward telemedicine, the use of telemedicine in Germany must be increased. This opens new perspectives for structurally weak regions and could help in reducing the burden in emergency departments.

## Abbreviations

eHealth: electronic health
mHealth: mobile health
